# Health professionals’ views on the barriers and enablers to evidence-based practice for acute stroke care: a systematic review

**DOI:** 10.1186/s13012-017-0599-3

**Published:** 2017-06-05

**Authors:** Leonard Baatiema, Michael E. Otim, George Mnatzaganian, Ama de-Graft Aikins, Judith Coombes, Shawn Somerset

**Affiliations:** 1 Regional Institute for Population Studies, University of Ghana, P.O Box LG96, Legon-Accra, Ghana; 2 School of Allied Health, Faculty of Health Sciences, Australian Catholic University, Sydney, Australia; 3 College of Health Sciences, University of Sharjah, Sharjah, United Arab Emirates; 4 College of Science, Health and Engineering, La Trobe Rural Health School, La Trobe University, Melbourne, Australia; 5 School of Pharmacy, University of Queensland, Brisbane, Australia

**Keywords:** Acute stroke, Evidence-based practice, Therapies, Services, Barriers, Enablers

## Abstract

**Background:**

Adoption of contemporary evidence-based guidelines for acute stroke management is often delayed due to a range of key enablers and barriers. Recent reviews on such barriers focus mainly on specific acute stroke therapies or generalised stroke care guidelines. This review examined the overall barriers and enablers, as perceived by health professionals which affect how evidence-based practice guidelines (stroke unit care, thrombolysis administration, aspirin usage and decompressive surgery) for acute stroke care are adopted in hospital settings.

**Methodology:**

A systematic search of databases was conducted using MEDLINE, Cumulative Index to Nursing and Allied Health Literature (CINAHL), Embase, PsycINFO, Cochrane Library and AMED (Allied and Complementary Medicine Database from 1990 to 2016. The population of interest included health professionals working clinically or in roles responsible for acute stroke care. There were no restrictions to the study designs. A quality appraisal tool for qualitative studies by the Joanna Briggs Institute and another for quantitative studies by the Centre for Evidence-Based Management were used in the present study. A recent checklist to classify barriers and enablers to health professionals’ adherence to evidence-based practice was also used.

**Results:**

Ten studies met the inclusion criteria out of a total of 9832 search results. The main barriers or enablers identified included poor organisational or institutional level support, health professionals’ limited skills or competence to use a particular therapy, low level of awareness, familiarity or confidence in the effectiveness of a particular evidence-based therapy, limited medical facilities to support evidence uptake, inadequate peer support among health professionals’, complex nature of some stroke care therapies or guidelines and patient level barriers.

**Conclusions:**

Despite considerable evidence supporting various specific therapies for stroke care, uptake of these therapies is compromised by barriers across organisational, patients, guideline interventions and health professionals’ domains. As a result, we recommend that future interventions and health policy directions should be informed by these findings in order to optimise uptake of best practice acute stroke care. Further studies from low- to middle-income countries are needed to understand the barriers and enablers in such settings.

**Trial registration:**

The review protocol was registered in the international prospective register of systematic reviews, PROSPERO 2015 (Registration Number: CRD42015023481)

**Electronic supplementary material:**

The online version of this article (doi:10.1186/s13012-017-0599-3) contains supplementary material, which is available to authorized users.

## Background

Translation of research evidence into clinical practice is a major imperative for health professionals in policy, management and research, worldwide. It is almost half a century since Cochrane challenged conventional health care practices which consequently paved the way for the present day evidence-based practice movement in medicine and health care [1]. Nonetheless, routine clinical practice still lags behind contemporary research evidence [2–4], despite international calls for research evidence to guide healthcare delivery [5]. Globally, there is no single solution to closing this knowledge to practice gap [4, 6, 7]. In general, it has been estimated to take about 17 years for research evidence to be translated into clinical practice [8]. Delays in the adoption of evidence-based practice could be attributed to a multiplicity of barriers [9–12] underpinned by a plethora of theoretical and conceptual perspectives [2, 3, 13, 14], which have emerged to shed light on these barriers or enablers.

Stroke is caused by an interruption or blockage in blood supply or arterial bleeding into or around the brain [15, 16]. The early stages (first 48 h) of an acute stroke are a critical time-window for appropriate interventions to either stop or slow down brain tissue decay and minimise mortality and morbidity [17]. To provide acute stroke care in the early stages, current recommendations from Level-1 evidence for best practice include (1) stroke care in a specialist stroke unit [18, 19], (2) thrombolytic therapy with intravenous tissue plasminogen activator (t-PA) within 4.5 h of an acute ischemic stroke [20–22], (3) aspirin administration within 48 h of acute ischemic stroke onset [23–25] and (4) decompressive surgery if required within 48 h of stroke onset [26, 27]. The use of t-PA for example is the most effective pharmacological therapy for acute ischemic stroke despite the persistence of controversies surrounding its usage. First reported by the National Institute of Neurological Disorders and Stroke (NINDS) trials with a treatment window of 3 h [22], a later trial extended the treatment time to 4.5 h upon acute ischemic stroke [21]. Given such evidence consistently showed sound clinical outcomes over time, clinical guidelines have been developed and continually updated to support the application of these interventions for improved patient outcomes [28–34]. However, despite this scientific evidence and increased support for their usage, translation into clinical practice is slow, and this is greatest in low–middle income countries [35–37]. For example, despite the net benefits associated with thrombolytic therapy for acute ischemic stroke, global uptake in low-income countries is about 3% compared to 50% in high-income countries [35]. A recent survey of acute stroke services in eleven major referral hospitals in Ghana also revealed the lack of use of t-PA for acute ischemic stroke care and the availability of only one stroke unit [38].

The reasons for the slow uptake remains poorly understood. Some studies have however attempted to shed light on such barriers, and these comprised inadequate medical facilities for acute stroke care, health professionals’ unwillingness for change, unawareness of evidence-based therapies, lack of health professionals’ competence to apply evidence-based therapies, limited staff capacity and decision-makers’ values and preferences could be attributed for the slow uptake [39–46]. Such barriers have resulted in the underutilisation of best practice interventions towards positive clinical outcomes. The recent Lancet series on Right Care [47], which seeks among others, to highlight the chronic underutilization of evidence-based interventions further underscores the centrality of this review.

To date, no study has attempted to systematically analyse published primary studies on the barriers and enablers perceived by health care professionals to influence the adoption of these four highly recommended acute stroke therapies or services. Prior studies on this topic were either limited in focus by only unilaterally exploring barriers related to the use of t-PA [41, 42, 44], neuroprotective therapy [45] or generalised acute stroke care guidelines [40]. A recent study by Craig et al. has also attempted to examine some of these barriers and enablers [48], though an important contribution, a different theoretical framework was used, and focused more on clinical behavioural components.

Our aim in this review was to identify health professionals’ views on the barriers and enablers to their use of the above recommended evidence-based acute stroke care interventions. An understanding of these barriers and enablers is important towards closing the current knowledge to practice gap in stroke clinical practice. With the increasing stroke burden in low–middle income regions in recent times [49–51] and where uptake levels of such interventions are presently lowest, a clearer understanding of barriers and enablers, primarily from such regions may also be essential in developing context-specific strategies to optimise uptake of evidence-based acute stroke care recommendations in clinical practice to improve patient outcomes.

## Methods

This review was conducted according to the Preferred Reporting Item for Systematic Reviews and Meta-Analysis (PRISMA) systematic review approach [52], as outlined in Additional file 1. The review protocol was registered in the international prospective register of systematic reviews, PROSPERO 2015 (Registration Number: CRD42015023481).

### Eligibility criteria

Studies based on the views of stroke specialists, medical doctors, nurses and allied health professionals were considered. Other health professionals including health managers, health planners, health policy-makers or any health executives’ about barriers or enablers to the uptake of evidence-based acute stroke care were included. For inclusion, interventions for evidence-based acute stroke care were restricted to barriers or enablers in relation to the provision of care in a stroke unit, thrombolytic therapy, the use of aspirin and decompressive surgery. Peer-reviewed articles of any study design were considered. Barriers and enablers based on database records were excluded. Included studies were based only on the views, opinions and experiences of the health professionals. Non-original research such as letters, commentaries, guidelines, magazines and editorials were excluded. Research studies with non-human components were also not considered.

### Search strategy

A systematic search of the literature was conducted electronically using MEDLINE, CINAHL, Embase, PsycINFO, Cochrane Library and AMED. Reference lists and bibliographies from eligible studies published from 1990 to 2016 were also considered for inclusion. The review considered studies published within this time duration to correspond with the period when evidence-based medicine movement and scholarship enjoyed renewed interest and acknowledgement [53]. This was also done to ensure included studies reflect current evidence of health professionals’ views on what acts as a barrier or an enabler to their uptake or adherence to evidence-based practice for acute stroke care. Due to lack of resources for language translation, all included studies were limited to studies published in English language. Search strings were designed to reflect related Medical Subject Heading (MeSH) terms, key terms and phrases from the selected databases related to the review aim. Details of the search terms used are presented in Additional file 2.

### Study selection

Results were downloaded and imported into EndNote for screening to first remove duplicates by one author (LB). The next stage involved the screening of the remaining studies based on the relevance of study titles and abstracts to the review aim. When articles had insufficient information in the title and abstract to support this screening, a full-text reading was conducted. This was followed by the selection of all potentially eligible studies in full text. A second author (SS) reviewed the selected full-text articles to ensure they met the eligibility criteria. Results of the full text were also shared with the remaining authors to validate, and none of the authors raised questions about their eligibility. Articles which met the inclusion criteria following full-text screening by two authors (LB and SS) were selected for the final analysis.

### Data extraction

A standardised data extraction tool (evidence table) was used to extract information relevant to the study aim by one author (LB). As shown in Table 1, the information extracted include the authors and year of publication, country of study, intervention, study aim, design, participants/sample, data collection methods and key findings on the barriers and enablers to uptake of acute stroke care interventions. This was systematically done to ensure extracted data characteristics from the eligible studies were consistent. The key findings and conclusions of the eligible studies which were reported as either barriers, enablers or barriers and enablers were identified by one author (LB). These findings were shared with the remaining reviewers to ensure consistency with the primary studies.

**Table 1:** Characteristics of included studies

Lead author, year and country	Study aim	Stroke intervention	Study design	Participants/sample size	Data collection methods tools	Barriers or enablers
Meurer (2011) [60] USA	To describe barriers to thrombolytic use in acute stroke care	t-PA	Qualitative study	- 65 emergency physicians - 62 nurses - 15 neurologists - 12 radiologists - 12 hospital administrators - 3 others (hospitalists and pharmacist)	Focus groups/interviews topic guide	Patient factors: delayed presentation, family issues, age of patient, demand for t-pa, language, adverse to taking ambulance, early symptom recognition Guideline factors: characteristics of the guideline, outcome expectancy, presence of contradictory guidelines or “position statements on guidelines, lack of clarity on guidelines Individual health professionals: lack of awareness of acute stroke guidelines, lack of guideline familiarity, interpretation confidence, lack of guideline agreement, lack of self-efficacy, lack of motivation, inadequate communication of the time sensitive nature of CT ordering and interpretation, inaccurate patient weight, staff recognition of stroke symptoms Resources and incentives: availability of scanner, financial issues, lack of motivation and ICU bed availability Organisation context/health system: lack of system process to alert radiologists of the emergency nature of stroke-related scans, laboratory-based barriers, limited neurosurgery, lack of follow up/feedback, ED overcrowding, lack of a protocol, limited neurology, hospital notification, lack of speed, pharmacy and drug delivery delay or shortage barriers, fear of liability for use or non-use of t-PA, triage barriers, difficulty arranging for transfer from clinics
Hargis (2015) [65] USA	To identify barriers to the administration of intravenous tissue plasminogen activator (t-PA)	t-PA	Cross-sectional study	Stroke coordinators (36)	Survey questionnaire	Individual health professionals: physician reluctance to use t-PA and lack of urgency in emergency department Professional interactions: poor communication between care providers Organisation context/health system: lack of a dedicated and trained stroke nurse, role definition not clear Patient factors: patients’ late arrival
Chan (2005) [63] USA	To assess the experience, knowledge and attitudes of emergency department directors on their use of t-PA	t-PA	Cross-sectional study	52 emergency physicians (directors)	Survey questionnaire	Guideline factors: ED directors’ attitudes regarding its safety Individual health professionals: suboptimal nursing staff support, lack of neurological support, lack of neurosurgical support, lack of radiological support, willingness to use t-PA to treat acute ischemic stroke Resources and incentives: lack of CT access and lack of CT interpretation availability Organisation context/health system: lack of institutional attitudes, availability of ED protocols for use t-PA use, general lack of institutional support, limited radiological back up for CT interpretation and lack of protocols to support t-PA use
O’Rourke (2013) [66] Australia	To determine stroke clinicians’ preferences for models of inpatient stroke unit care and perceived barriers to establishing a comprehensive stroke unit model	Stroke unit	Cross-sectional study	228 participants −99 allied health −72 nurses −57 doctors	Survey questionnaire	Organisation context/health system: Stroke unit care not seen as priority by hospital, lack of management support and lack of staffing Professional Interactions: lack of allied health support, lack of nursing support and lack of physician support Resources and incentives: lack of time, lack of money and lack of physical space Guideline factors: lack of evidence
William (2013) [69] Australia	To identify barriers which prevent rural health care providers from utilising t-PA in acute ischaemic stroke and proposes possible support mechanisms to increase its utilisation	t-PA	Cross-sectional study	11 physicians 13 nurses	Survey questionnaire	Organisation context/health system: lack of stroke protocols and pathways support, lack of administrative support, emergency department delays, lack of support Resources and incentives: unsuitable hospital setting Individual health professionals: lack of knowledge and education on the use of t-PA, uncertainty with patient selection criteria, experience with t-PA inclusion and exclusions, personal stroke neurological experience, clinical diagnostic uncertainty Patient factors: pre-hospital delays Guideline factors: risk of intracerebral haemorrhage, uncertainty about benefits of t-PA
Purvis (2014) [62] Australia	To determine the local enablers and barriers to providing evidence-based stroke care	Stroke unit and t-PA	Qualitative study	84 clinicians (nurses, allied health staff, department or unit managers and physicians)	Semi-structured interviews and focus group topic guide	Organisation context/health system: shortage of neurologists, lack of formalized guidelines or protocols, inconsistent use of pathways, lack of staff to constantly update pathways/guidelines, lack of formalised process to support consistent education, heavy workloads, lack of dedicated physician or nurse for stroke care, limited funding for staff professional development, lack of dedicated allied health positions, frequent rotation of staff, lack of executive support and employment of part time staff Resources and incentives: limited number of stroke unit beds, lack of dedicated stroke unit, lack of resources, lack of time to provide education Professional interactions: lack of strong medical leadership–delays in clinical decisions Individual health professionals: resistance from ED doctors to use thrombolysis, lack of awareness on time constraints to t-PA, inability to administer t-PA and inconsistency administering of t-PA
Grady (2014) [64] Australia	To assess emergency physicians’ perceptions of individual and system enablers to the use of tissue Plasminogen activator in acute stroke	t-PA	Cross-sectional study	429 participants Australasian College for Emergency Medicine Members	Survey questionnaire	Organisation context/health system: performance monitoring, providing feedback on stroke care performance, checklist/decision aids (maintenance) Individual health professionals: knowledge on the use of t-PA treatment, skill and competency to use t-PA, modelling use of t-PA by senior staff
Van Der Weijden (2004) [68] The Netherlands	To investigate barriers for guideline adherence to bring about suggestions for possible implementation strategies	Aspirin t-PA	Cross-sectional study	201 neurologists	Survey questionnaire	Organisation context/health system: lack of manpower, poor patient flow to the rehabilitation care centre and time to treatment delays (defiant referral behaviour of general practitioners) Resources and incentives : insufficient hospital logistics or beds Individual health professionals: negative attitude towards guideline use, lack of experience or competence, lack of knowledge Patient factors: patients are too late in hospital, hesitation by the patient or carer to use guideline Guideline factors: lack of confidence in the evidence, fear of complications, disagreement with stroke care guidelines and doubt of cost effectiveness/high cost of guideline implementation
Slot (2009) [67] Scandinavian countries (Norway, Denmark and Sweden)	To describe the use of t-PA in the hospitals, assess stroke doctors’ opinions on the use of t-PA, identify existing barriers against treatment and to ways to overcome the barriers	t-PA	Cross-sectional study	453 doctors	Survey questionnaire	Organisation context/health system: lack of urgent triaging of stroke patients by ED due to high workload, hospitals lack of good protocols⁄routines for t-PA, and ambulance service staff inadequate triaging of acute stroke patients Individual health professionals: ambulance staff lack of knowledge about t-PA, ED lack of knowledge about thrombolytic treatment and disapproval of the use of t-PA by physicians Patient factors: patients ⁄lack of early recognition of symptoms, patients delay in contacting ambulance service and patients disinterest in t-PA due to side effects Guideline factors: risk of intracranial haemorrhage
Stecksen (2013) [61] Sweden	Identify facilitators of and barriers to the implementation of national guidelines on thrombolytic therapy for acute ischemic stroke	t-PA	Qualitative study	9 physicians 7 nurses	Semi-structured interviews Interview guide	Organisation context/health system: stressful and overburdened working conditions, formal power structures, failure to react to guideline deviations, limited human resource capacity/few staff for stroke care, lack of continuity, duty schedule inhibiting training and lack of institutional support Resources and incentives: limited financial resources and insufficient time Professional interactions: poor professional identity, insufficient recognition by peers, inter-intra professional power structures, lack of support from more advanced hospitals and prestige and power relations Individual health professionals old-fashioned views, lack of experience with thrombolytic therapy, limited time, patients’ recruitment difficulties, lack of knowledge, lack of awareness of stroke as an emergency by ambulance services and other hospital staff and anxiety in using t-PA Patient factors: low awareness/knowledge of stroke symptoms causes delays Guideline factors: low expectations of therapeutic options, undue respect for the treatment (t-PA) and strict criteria for t-PA

###  Data synthesis

Data analysis involved a thematic analysis of the results from the eligible studies. Based on the Tailored Implementation for Chronic Diseases project [54], a pre-existing framework of seven domains developed by implementation science researchers to examine what informs change in clinical practice [55] was followed to categorise the themes of barriers and enablers. The checklist of seven domains comprised guideline factors, individual health professionals’ factors, patient factors, professional interactions, incentives and resources, capacity for organisational change, and social, political and legal affairs. Additional file 3 provides further explanation of each domain. This process was done by one reviewer (LB) who is experienced in categorising themes using pre-existing frameworks. This was done with constant reference to the content of the pre-existing framework and identified barriers and enablers from the articles to ensure appropriate classification. Another reviewer (SS) validated the classification of the barriers and enablers (See Table 2), and one disagreement was recorded during this stage but was quickly resolved in consultation with another author (AdGA). One author (LB) consequently weighted each domain of barriers/enablers in a tabular form (See Table 3) according to the frequency of each barrier or enabler as reported in the articles.

**Table 2:** Domain of barriers or enablers to evidence uptake

Author and year	Stroke therapy or intervention	Guideline factors	Individual health professionals	Patient factors	Professional interactions	Incentives and resources	Capacity for organisational change	Social, political and legal factors
O’Rourke (2013) [66]	Stroke unit	x			x	x	x	
Grady (2014) [64]	Thrombolysis		x		x		x	
William (2013) [69]	Thrombolysis	x	x	x			x	
Van Der (2004) [68]	Aspirin and thrombolysis	x	x	x		x	x	
Slot (2009) [67]	Thrombolysis	x	x	x		x	x	
Meurer (2011) [60]	Thrombolysis	x	x	x		x	x	
Purvis (2014) [62]	Stroke unit and thrombolysis	x	x	x	x	x	x	
Stecksén (2013) [61]		x	x		x	x	x	
Hargis (2015) [65]	Thrombolysis	x	x	x	x	x	x	
Chan (2005) [63]	Thrombolysis	x	x			x	x	

X indicates a particular thematic barrier or enabler reported by the author (s)

### Assessment of methodological quality

To capture the unique reporting differences within qualitative and quantitative studies, two separate quality reporting assessment tools were used. The checklist by the Joanna Briggs Institute for assessing qualitative studies was used for the qualitative studies [56], while the guidelines suggested by the Centre for Evidence-Based Management to appraise surveys was also used for the quantitative studies [57]. These checklists were used because they have comprehensively clear score sheets and instructions which enabled the authors to assess the relevance and rigour of all included studies. Given that there is still lack of consensus on the criteria for assessing the quality of qualitative studies in systematic reviews [58, 59]; included qualitative studies were not based on their quality scoring but on the basis of their overall contribution to the synthesis rigour. One reviewer (LB) appraised the quality of included studies. Another reviewer (SS) carried out a separate rating and slight variations were observed. However, these differences were quickly addressed by the two reviewers.

## Results

### Study selection

**Fig. 1:**
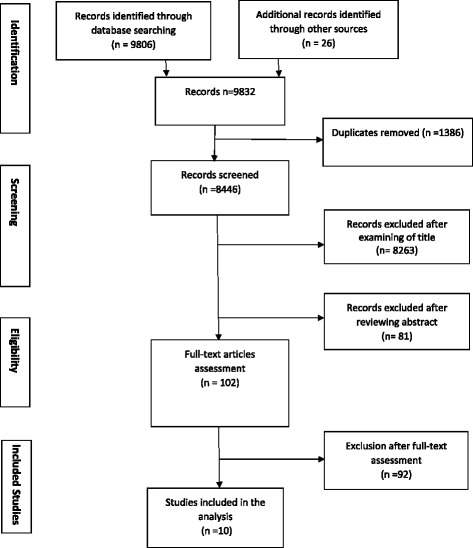
Flow chart on selection and screening process for eligible studies using the PRISMA methods

The electronic search yielded 9832 studies [MEDLINE = 2518, CINAHL = 458, AMED = 221, PscINFO = 1229, Embase = 873, Cochrane Library = 4507 and 26 additional studies retrieved from other sources]. After removing 1386 duplicates, 8446 studies remained. Screening based on title and abstract relevance excluded 8263 and 81articles, respectively. Studies excluded at this stage were either due to the fact that they were not primary studies, had irrelevant topics, that is, not focused on barriers and enablers to the four recommended evidence-based stroke care interventions. Other reasons for exclusion include duplicate studies, letters and editorials. A full-text screening of the remaining 102 potentially eligible studies led to further exclusions of 92 studies as they were deemed irrelevant to the study aim, focused on different population of interest, included review papers, guidelines and case reports. Overall, 10 studies met the inclusion criteria (See Fig. 1).

### Study characteristics

Three qualitative [60–62] and seven quantitative studies [63–69] were included. Quantitative studies employed online and postal surveys while the qualitative studies used semi-structured interviews and focus group methods. Whereas analysis of the quantitative studies was conducted using predominantly descriptive statistics, thematic analysis guided the analyses of the qualitative studies. The total number of included participants was 1692, and these comprised nurses, general medical doctors, neurologists, emergency department physicians, allied health staff and health managers. Included studies were published between 2004 and 2015. Four studies were conducted in Australia [62, 64, 66, 69], three in the USA [60, 63, 65], two in Sweden [61, 67], and one each in Norway [67], Denmark [67] and the Netherlands [68]. Most of the barriers or enablers identified in the quantitative studies were also found in the qualitative studies. Studies predominantly examined the barriers or enablers to the use of thrombolysis [60, 61, 63–65, 67, 69]. One study focused exclusively on barriers related to the establishment of a stroke unit [66], another on the uptake of both aspirin and thrombolysis [68] and the remaining on stroke unit and thrombolysis [62]. Although most of the eligible studies focused on barriers related to the use of evidence-based care for acute stroke, all included studies reported on three or more related barriers or enablers (See Table 1 for additional information).

### Quality assessment

The overall quality of the quantitative studies was moderate given that certain methodological limitations were found in the eligible studies. Only one study described in detail the sampling of study subjects and employed sampling techniques to minimise selection bias [69]. However, the rest of the cross sectional studies did not include substantive information on sampling techniques used to minimise selection bias. Three of the eligible studies reported high response rates of 91 [63], 92 [67] and 95.8% [66]. Conversely, low response rates of 13 [64] and 26% [69] were also noted. Details of quality of reporting evaluations are presented in Table 4.

**Table 3:** Barriers and enablers to evidence-based acute stroke care

Domain of barriers and enablers	Frequency (%)
Guideline factors	16 (10.38%)
Individual health professionals	39 (25.32%)
Patient factors	15 (9.74%)
Professional interactions	10 (6.49%)
Incentives and resources	17 (11.03%)
Capacity for organisational change	57 (37.01%)
Social, political and legal factors	0 (0.0%)

Note: The weighted frequency was calculated based on the number of times a particular barrier or enabler was reported in the eligible studies

On the other hand, none one of the eligible qualitative studies reported on theoretical or philosophical bases for methodological choice, limiting the ability to situate and assess methodological relevance. However, there was a common approach to the reporting of specific data collection and the analysis process. However, none of the studies reported on how the philosophical paradigm influenced data analysis and interpretation. All qualitative studies adequately described how interviews were conducted, although no assessment data trustworthiness through triangulation or member checking was reported. Finally, two of the qualitative studies [48, 62] addressed the issue of reflexivity, that is, potential reporting bias related to the researcher’s professional background or areas of interest.

### Synthesis of results

#### Summary of evidence: main barriers and enablers to evidence uptake

Overall, four studies reported on both barriers and enablers to uptake of evidence-based acute stroke therapies [61, 62, 66, 69] whilst five reported on only barriers to evidence uptake [60, 63, 65, 67, 67] and one study had an explicit focus on enablers to uptake of evidence-based care for acute stroke [63]. Despite some studies reporting on both barriers and enablers, studies which focused only on barriers often made reference to or inferred enablers as the opposite of the barriers, an approach that has been adopted in the present review. Thus, barriers and enablers were analysed and discussed collectively. As reported below, Table 3 shows the distribution and weighted frequency of each barrier which provides information on the potential significance of each barrier and enabler to the uptake of the four recommended acute stroke care interventions.

**Table 4:** Critical appraisal of eligible studies

	Appraisal questions for survey	O’Rourke (2013) [66]	William (2013) [69]	Van Der Weijden (2004) [67]	Grady (2014) [64]	Hargis (2015) [65]	Chan (2005) [63]	Slot (2009) [67]
1	Did the study address a clearly focused question/issue?	Y	Y	Y	Y	Y	Y	Y
2	Is the research method (study design) appropriate for answering the research question?	Y	Y	Y	Y	Y	Y	Y
3	Is the method of selection of the subjects (employees, teams, divisions, organisations) clearly described?	U	Y	Y	Y	Y	N	Y
4	Could the way the sample was obtained introduce (selection) bias?	N	N	N	N	U	N	N
5	Was the sample of subjects representative with regard to the population to which the findings will be referred?	N	N	N	Y	N	N	Y
6	Was the sample size based on pre-study considerations of statistical power?	N	Y	Y	N	N	N	N
7	Was a satisfactory response rate achieved?	Y	N	N	N	Y	Y	Y
8	Are the measurements (questionnaires) likely to be valid and reliable?	N	Y	N	Y	N	N	N
9	Was the statistical significance assessed?	N	N	N	Y	N	N	N
10	Are confidence intervals given for the main results?	N	N	Y	N	N	N	N
11	Could there be confounding factors that haven’t been accounted for?	N	N	N	N	N	N	U
12	Can the results be applied to your organisation?	N	U	U	N	N	N	Y
	Yes (Y), Can’t Tell (U) and NO (N)							
	Critical Appraisal Questions for Qualitative Studies	Meurer (2011) [60]	Purvis (2014) [62]	Stecksén (2013) [61]				
1	Is there a congruity between the stated philosophical perspective and the research methodology?	N	N	N				
2	Is there a congruity between the research methodology and the research question or objectives?	Y	Y	Y				
3	Is there a congruity between the research methodology and the methods used to collect the data?	Y	Y	Y				
4	Is there a congruity between the research methodology and the representation and analysis of data?	Y	Y	Y				
5	Is there a congruity between the research methodology and the interpretation of results?	Y	Y	Y				
6	Is there a statement locating the researcher culturally or theoretically?	Y	Y	N				
7	Is the influence of the researcher on the research and vice versa addressed?	U	Y	U				
8	Are participants, and their voices, adequately represented?	Y	Y	N				
9	Is the research ethical according to current criteria or, for recent studies, is there evidence of ethical approval by an appropriate body?	Y	Y	Y				
10	Do the conclusions drawn in the research report flow from the analysis, or interpretation, of the data?	Y	Y	Y				
	Yes (Y) No (N) Unclear (U) Not Applicable (NA)							

#### Capacity for organisational change

This category of barriers/enablers was the most highly cited by participants in all the eligible studies. According to the health professionals, the use of evidence-based care could be challenged by lack of institutional support [61]. They further highlighted limited health staff capacity especially lack of a stroke nurse or specialist [60, 62, 65] and inadequate funding opportunities for staff professional development [62, 64]. For example, participants reported that some hospitals were unable to provide or formalise acute stroke care guidelines to facilitate health staff use of evidence-based therapies [60, 69]. Additionally, instances were cited where there was limited or no executive support for professional development or upgrading to deliver current therapies for acute stroke according to best scientific evidence [62]. Of the varied barriers reported under this category, workload demands were also commonly cited as a key hindrance to the implementation of evidence-based acute stroke care [60, 62, 67]. In one study [69], 71% of participants indicated lack of protocols and pathways. The study by Van der Weijden et al. identified organisational level barriers as the most significant barriers to uptake of evidence-based practice [68].

#### Individual health professionals

Individual health professionals’ factors were reported by participants as important barriers/enablers from the eligible studies. This domain of barriers was found in nine included studies [60–65, 67–69]. In the views of most participants, uptake of evidence-based interventions such as thrombolytic therapy is slow or not happening due to health professional’s lack of awareness of a particular intervention [60, 61, 68, 69], lack of skills or self-efficacy to apply the intervention [60, 61, 68] or low motivation to implement an evidence-based therapy [60]. For example, in one study, 50% of participants indicated their lack of knowledge on the use of thrombolytic therapy hampered uptake in their routine clinical practice [60]. They also outlined barriers such as old-fashioned views about some specific acute stroke therapies [61]. Further, one study [63] reported that some neurologists disapprove of the use of thrombolytic therapy, which was agreed by (33%) of respondents.

#### Resources and incentives

This was another major domain of barriers or enablers to evidence uptake for acute stroke care. A total of eight of ten eligible studies identified resources and incentives related barriers/enablers as crucial to evidence uptake [60–63, 65–68]. Some of the common barriers/enablers comprised limited physical space to establish stroke units [66], lack of CT scans [63], lack of financial resources [61, 62, 66, 68], limited time [61, 66], limited stroke beds [62, 66] and limited staff capacity [61–63, 66, 68]. These factors were common in both qualitative and quantitative studies in this review.

#### Guidelines factors

The present review has shown the nature and characteristics of specific evidence-based therapies for acute stroke could influence their levels of uptake. Nine of ten eligible studies reported barriers related to the characteristics or the nature of evidence related to the stroke intervention or guidelines [60–63, 65–69]. Views related more to health professionals’ misconceptions about the level of effectiveness of some acute stroke care therapies such as thrombolysis. For example, despite evidence that the benefits of thrombolysis outweigh potential associated side effects, participants expressed doubts in the effectiveness of this therapy because they were concerned about severe bleeding and other complications. In one study [69], 73% of respondents indicated risk of symptomatic intracerebral haemorrhage as a key barrier to administering thrombolysis. In another study [63], 33% of the participants expressed uncertainty about the evidence of using thrombolytic therapy for acute ischemic stroke and recommended the need for further studies for definitive evidence of its efficacy before they would use it for patient care. Disagreement on the recommended dosage for aspirin was also highlighted by participants in one study [68].

#### Patient factors

Within this domain of barriers and enablers, six studies highlighted factors such as late arrival to seek care, patients’ or relatives’ lack of awareness of early stroke symptoms or patients’ decision for other acute care interventions outside the standardised recommendation [60, 62, 66, 67–69]. The most frequently reported patient-related barrier was patients’ late arrival in emergency departments to receive thrombolysis. For example, one study [69] reported that 91% of respondents indicated patients’ late arrival for acute care as the major barrier. Another study ranked delayed patient presentation for care as the major barrier to the use of thrombolytic therapy [65] due to the patients’ failure to recognise stroke symptoms. Another key barrier was patients’ preference for the non-use of thrombolysis as a therapeutic option due to perceived side-effects of this treatment option [67].

#### Professional interactions

The uptake of evidence-based care for acute stroke can also be influenced by the form and nature of interactions among health professionals, especially engagement with clinical leaders. Five studies showed evidence of this domain of barriers/enablers [61, 62, 64–66]. The present review found this as among the least described barriers/enablers in the included studies. Barriers suggested by participants included: inadequate communication especially among clinical staff [65], lack of clinical leadership or support from senior clinicians [62]. As an example, Hargis et al. reported that 14% of respondents cited poor communication between emergency department staff, and the neurology team affected the use of thrombolytic therapy [65].

## Discussion

This review aimed to explore the main barriers/enablers underlying adoption of evidence-based therapies for treatment and management of acute stroke. To date, prior studies have focused largely on barriers or enablers to generalised acute stroke guidelines or thrombolysis specifically. This review addressed a knowledge gap on the main barriers or enablers to the uptake of the four recommended evidence-based therapies/service for acute stroke, namely, stroke unit care, thrombolytic therapy, aspirin and decompressive surgery.

The specific innovations of this review are its primary focus on the four recommended evidence-based therapies for acute stroke care and the inclusion of both quantitative and qualitative study designs, both of which add depth to the analysis. Although this review was limited to ten eligible studies, there seems to be a saturation of potential determinants given the commonality and recurrence of barriers and enablers revealed between studies. There was also a significant overlap in the reported barriers or enablers, although these characterisations differed between health professionals. Findings from prior reviews on barriers to thrombolysis uptake [41, 44, 45, 70], other studies on the barriers and enablers to triaging, treatment and patients’ transfer in emergency departments (ED) [48] and adherence to general stroke clinical guidelines [40], corroborated with majority of the barriers/enablers identified in this review.

On the most important barriers or enablers from the present review, organisational context or structural level factors were the most cited barriers or enablers to uptake of evidence-based care for acute stroke by health professionals. This finding substantiates the results of earlier works [9, 41, 70]. Given the importance attached to this category of barriers and as reflected in earlier works, a greater effort to address these barriers should be prioritise by health managers and planners for optimal uptake of evidence-based practice. Further, consistent with the literature [9, 40, 41, 45, 71], the barriers related to the individual health professional and guideline level barriers, availability of adequate health resources and medical facilities were also predominant in this review.

The barriers/enablers associated with social, political and legal factors were not reported by any of the eligible studies, thus leaving a gap in our understanding of whether such thematic barriers or enablers play any important role in evidence-based care uptake. It is plausible that their influence on evidence-uptake is negligible and may not warrant immediate attention of health policy-makers and health managers. The absence of evidence for this domain of barriers/enablers in this review was also evident in the checklist employed to contextualise the discussion in this review. In that review [55], which promulgated the checklist, this particular domain attracted the least eligible studies.

Importantly, the eligible studies were all conducted in high-income countries and so the findings may not be directly relevant to those in low–middle income countries. The inadequacy of medical facilities, limited health staff capacity and other health resource constraints characterised in low- and middle-income countries may emerge as the most important barrier since health systems in these contexts always have fewer resources overall compared to high-income countries.

This review has also underscored the need for increased attention on patient level barriers. Specifically, patients’ late arrival in ED settings for care because of lack of recognition of early stroke symptoms was notable. To address the low awareness or lack of early recognition of stroke symptoms, we recommend the need for increased public health campaigns and research emphasising the urgent need to seek care at stroke symptom onset, as highlighted by the ‘time is brain’ research study [72] and the ‘FAST’ stroke awareness campaign messages in the UK [73, 74]. The UK FAST stroke awareness campaign strategy could be a unique exemplar for low- and middle-income countries where evidence [75–77] suggest low awareness of stroke symptoms is a major obstacle to care. With the exception of thrombolytic therapy, the barriers or enablers on the remaining three evidence-based recommendations were less explored. No studies explored decompression surgery, although an earlier review suggested patient level barriers as more essential [45]. Other researchers have cited limited access to computed tomographic (CT) brain scans in low-middle income as the most important factors to address to improve uptake of aspirin therapy [78].

### Implications

The analysis from this review may inform the circumstances in which health professionals are able to provide evidence-based care for acute stroke patients. Despite the increased scholarship and policy recommendations for this, the reported barriers or enablers persist, consequently depriving patients of sound and effective therapies. Given that previous evidence suggest, overall, a significant number of patients receive clinical care without sound scientific evidence [2, 10, 79], these findings have the potential to contribute to present efforts aimed at ensuring stroke patients receive effective care.

Increasingly, reports of the rising incidence and mortality rates from stroke in low- and middle-income countries continue to attract the attention of global health authorities. Nonetheless, studies thus far have indicated a low uptake of evidence-based care for acute stroke in Africa and other low/middle income regions [35, 36]. However, no eligible studies were found in low- and middle-income countries to improve understanding about the factors accounting for this apparent gap. It is essential to explore the barriers or enablers in the context of Africa and other low- and middle-income regions to develop context-specific interventions to enhance uptake of evidence-based care for acute stroke.

Various health professionals play major primary roles as acute caregivers and consequently have unique challenges that deserve attention in future studies since this review was unable to separate determinants according to specific health professionals. Future research should endeavour to explore the barriers or enablers unique to stroke specialists, medical doctors, nurses and allied health staff. As emphasised earlier, identifying the views of stroke patients and carers on the barriers and enablers to stroke care should be part of future research efforts.

### Strengths and limitations

An important strength of this review is its primary focus on the four recommended evidence-based care interventions. The inclusion of both quantitative and qualitative study designs further adds to the analytical breadth and depth of this review. Nonetheless, this review has some limitations. First, we acknowledge that since this study was limited to studies published in English language, there remains a possibility other relevant studies and insights from LMIC were missed. Also, the screening process for eligible studies was conducted by a single author, and this may have affected the accuracy, reliability and transparency of the process. Additionally, the search for relevant studies was limited to only peer-reviewed journals thus potentially relevant theses, conference presentations and book chapters were excluded. Although, the reasons for the lack of studies from low- and middle-income countries remains unclear, this could be explained by the prevailing situation of limited international literature on the uptake of evidence-based acute stroke care interventions from such settings.

The limited number of eligible studies made it impossible to draw definitive conclusions about the primary barriers or enablers to evidence uptake for acute stroke care. Also, although the present study attempted to rank the importance of the barriers and enablers based on their weighted frequencies, this is not optimal. This field is less developed with currently no time-tested approaches to qualitatively rate the importance of such drivers to change in healthcare. Approaches such as the GRADE-CERQual framework to measure the confidence of synthesised evidence [80] could be explored in similar reviews in future. As we used a pre-designed taxonomy of barriers and enablers to contextualise our findings, it is possible other relevant barriers and enablers considered unfit to the framework were inadvertently missed out.

## Conclusions

The reported barriers or enablers mapped well with the previously proposed taxonomy of barriers or enablers. Our findings are consistent with previous studies [9, 40] where lack of adherence to or inadequate use of evidence-based care was attributed to organisational level factors, professionals’ lack of awareness and familiarity to a particular evidence-based care, financial constraints, lack of confidence in a particular therapy, fear of adverse effects, personal beliefs, patient delays, lack of time to implement evidence-based treatment guidelines and preferences or values about the use of evidence-based care.

Despite considerable effective therapeutic options for acute stroke care, poor understanding of barriers or enablers and lack of a clear evidence-based health policy to ensure their uptake render such therapeutic services underutilised. In light of this, efforts by health managers and policy-makers to formulate context-specific policies and design interventions to enhance uptake of evidence-based care should be informed by these barriers and enablers. Following this review, we are also proposing research studies be conducted in low-middle income countries to enhance our understanding of the key barriers accounting for the currently low uptake levels of evidence-based acute stroke care interventions.

## Additional files

Additional file 1: PRISMA 2009 Checklist. (DOCX 15 kb)

Additional file 2: Search strategy for MEDLINE. (DOCX 17kb)

Additional file 3: Checklist for Barriers and Enablers. (DOCX 12 kb)

## Abbreviations

AMED: Allied and Complementary Medicine Database
CINAHL: Cumulative Index to Nursing and Allied Health Literature
CT: Computed tomographic
ED: Emergency department
FAST: Facial drooping, arm weakness, speech difficulties and time
GRADE-CERQual: Confidence in the Evidence from Reviews of Qualitative Research
ICU: Intensive care unit
MeSH: Medical Subject Heading
NINDS: National Institute of Neurological Disorders and Stroke
PRISMA: Preferred Reporting Item for Systematic Reviews and Meta-Analysis
t-PA: Intravenous tissue plasminogen activator
