# Supplementary material for: Health professionals’ views on the barriers and enablers to evidence-based practice for acute stroke care: a systematic review
Source: Implement Sci. 2017 Jun 5;12:74. doi: 10.1186/s13012-017-0599-3 (PMC5460544; doi:10.1186/s13012-017-0599-3)
Supplement: Supplementary file 2 — Search strategy for MEDLINE. (DOCX 17kb) [file 13012_2017_599_MOESM2_ESM.docx]

**Additional File 2_** **Search Strategy for Medline**

| **Search** | **Query** |
| --- | --- |
| #1 | (Stroke) OR (Acute Stroke) OR (Cerebrovascular Disease) OR (Cerebrovascular Accident) OR (CVA) OR (Brain Attack) OR (Cerebral Infarction) OR (Lacunar ) OR (Chronic Stroke ) OR (Neurological Disorder) OR (Brain Tumour) OR (Brain Accident) OR (Brain Vascular Accident) OR (Cerebral Vascular Accident) OR ( Cerebrovascular Trauma) OR (Cerebrovascular Injury ) OR (Cerebral Infarction) |
| #2 | (Stroke unit) OR (Organised stroke care) OR (stroke ward) OR (Organised in-patient care) OR (Organised Care) OR (Stroke Service) OR (Multidisciplinary Stroke Care) OR (Multidisciplinary Stroke Team) OR (Stroke Treatment Team) OR (Stroke Management Team) |
| #3 | (Aspirin) OR (Antiplatelet) OR (Fibrinolytic Agents) OR (Acetylsalicylic Acid) OR (Antithrombocytic Agent) |
| #4 | (Thrombolytic Therapy) OR (Tissue Plasminogen Activator) OR (t-PA) OR (rt-PA) OR (Alteplase) OR (Thrombolysis) OR (Intravenous (IV) OR (Blood Clot Lysis) OR (Fibrinolytic Therapy) OR (Brain Ischemia drug therapy) OR (Fibrinolytic Agents therapeutic use) OR (Stroke drug therapy) OR (Thrombolytic Therapy methods) |
| #5 | (Hemicraniectomy) OR (Decompressive surgery) OR (Decompressive Craniectomy) OR (Neuroprotective Therapy) OR (Vascular Surgery) OR (Neurosurgery) |
| #6 | (Barrier*) OR (facilitat*) OR (enabl*) OR (inhibit*) OR (imped*) OR (challenges) OR (obstacles) OR (utilis*) OR (uptake) OR (implement*) OR (translat*) OR (compliance) OR (adherence) OR (adhere*) |
| # 7 | S2 OR S3 OR S4 OR S5 |
| #8 | S1 AND S7 |
| #9 | S6 AND S8 |
| Limiters | Full Text and Abstract  Date of Publication: 1990-2016  English Language  Research Article  Human  Academic Journals |
