# Supplementary material for: Health professionals’ views on the barriers and enablers to evidence-based practice for acute stroke care: a systematic review
Source: Implement Sci. 2017 Jun 5;12:74. doi: 10.1186/s13012-017-0599-3 (PMC5460544; doi:10.1186/s13012-017-0599-3)
Supplement: Supplementary file 3 — Checklist for Barriers and Enablers. (DOCX 12 kb) [file 13012_2017_599_MOESM3_ESM.docx]

**Additional File 3: Checklist for Barriers and Enablers**

| **Domains** | **Barriers and Enablers to Evidence-based Practice** |
| --- | --- |
| Guideline Factors | These describe barriers related to the characteristic of the acute stroke intervention in terms of its user-friendliness, applicability, access, cost, evidence effectiveness level, cost-effectiveness, complexity, adaptability and compatibility. |
| Individual Health Professionals | This comprised factors such as health professionals’ competence, skill, knowledge, familiarity and agreement levels to use a particular therapy. Their preferences, concerns or level of trust, attitudes, values, and motivations are also important factors in this category. |
| Patient  Factors | Patient level drivers to evidence-uptake include their compliance to a particular therapy, patient-health professional interaction, patient delays in seeking care due to late arrival or low awareness of stroke symptoms. |
| Professional Interactions | Here, we refer to the level of peer or team support or discouragement, collaborations or networks, mutual trust, level of communication with team members all fall under this domain of barriers or enablers |
| Incentives and Resources | Incentives and resources encompass availability of medical facilities to support evidence-uptake. These include CT scan availability, specialist stroke nurse or a physician, time, working space, funding issues, availability of incentives for staff, among others. |
| Capacity for Organisational Change | This category considers organisational level factors such as supporting policies for evidence implementation, receptive organisational context, provision and availability of guidelines for staff use, process of referral, organisational leadership, institutional structures, workload and staffing levels |
| Social, Political and Legal Factors | The comprised the existence or non-existence of regulatory and legal frameworks or policies supporting or against evidence uptake. |
